# Molecular Evolution of the Capsid Gene in Norovirus Genogroup I

**DOI:** 10.1038/srep13806

**Published:** 2015-09-04

**Authors:** Miho Kobayashi, Shima Yoshizumi, Sayaka Kogawa, Tomoko Takahashi, Yo Ueki, Michiyo Shinohara, Fuminori Mizukoshi, Hiroyuki Tsukagoshi, Yoshiko Sasaki, Rieko Suzuki, Hideaki Shimizu, Akira Iwakiri, Nobuhiko Okabe, Komei Shirabe, Hiroto Shinomiya, Kunihisa Kozawa, Hideki Kusunoki, Akihide Ryo, Makoto Kuroda, Kazuhiko Katayama, Hirokazu Kimura

**Affiliations:** 1 Gunma Prefectural Institute of Public Health and Environmental Science, Maebashi-shi, Gunma 371-0052, Japan; 2 Hokkaido Institute of Public Health, Sapporo-shi, Hokkaido 060-0819, Japan; 3 Aomori Prefectural Public Health and Environment Center, Aomori-shi, Aomori 030-8566, Japan; 4 Iwate Prefectural Meat Inspection Center, Shiwa-cho, Iwate 020-3311, Japan; 5 Miyagi Prefectural Institute of Public Health and Environment, Sendai-shi, Miyagi 983-0836, Japan; 6 Saitama Institute of Public Health, Yoshimi-machi, Saitama 355-0133, Japan; 7 Tochigi Prefectural Institute of Public Health and Environmental Science, Utsunomiya-shi, Tochigi 329-1196, Japan; 8 Kanagawa Prefectural Institute of Public Health, Chigasaki-shi, Kanagawa 253-0087, Japan; 9 Kawasaki City Institute for Public Health, Kawasaki-shi, Kanagawa 210-0821, Japan; 10 Miyazaki Prefecture Kobayashi Meat Inspection Center, Kobayashi-shi, Miyazaki 886-0004, Japan; 11 Yamaguchi Prefectural Institute of Public Health and Environment, Yamaguchi-shi, Yamaguchi 753-0821, Japan; 12 Ehime Prefectural Institute of Public Health and Environmental Science, Matsuyama-shi, Ehime 790-0003, Japan; 13 Department of Safety Research on Blood and Biological Products, Yokohama City University Graduate School of Medicine, Yokohama-shi, Kanagawa 236-0004, Japan; 14 Department of Molecular Biodefence Research, Yokohama City University Graduate School of Medicine, Yokohama-shi, Kanagawa 236-0004, Japan; 15 Pathogen Genomics Center, National Institute of Infectious Diseases, Musashimurayama-shi, Tokyo 208-0011, Japan; 16 Department of Virology II, National Institute of Infectious Diseases, Musashimurayama-shi, Tokyo 208-0011, Japan; 17 Infectious Disease Surveillance Center, National Institute of Infectious Diseases, Musashimurayama-shi, Tokyo 208-0011, Japan

## Abstract

We studied the molecular evolution of the capsid gene in all genotypes (genotypes 1–9) of human norovirus (NoV) genogroup I. The evolutionary time scale and rate were estimated by the Bayesian Markov chain Monte Carlo (MCMC) method. We also performed selective pressure analysis and B-cell linear epitope prediction in the deduced NoV GI capsid protein. Furthermore, we analysed the effective population size of the virus using Bayesian skyline plot (BSP) analysis. A phylogenetic tree by MCMC showed that NoV GI diverged from the common ancestor of NoV GII, GIII, and GIV approximately 2,800 years ago with rapid evolution (about 10^−3^ substitutions/site/year). Some positive selection sites and over 400 negative selection sites were estimated in the deduced capsid protein. Many epitopes were estimated in the deduced virus capsid proteins. An epitope of GI.1 may be associated with histo-blood group antigen binding sites (Ser377, Pro378, and Ser380). Moreover, BSP suggested that the adaptation of NoV GI strains to humans was affected by natural selection. The results suggested that NoV GI strains evolved rapidly and date back to many years ago. Additionally, the virus may have undergone locally affected natural selection in the host resulting in its adaptation to humans.

Norovirus (NoV) of the genus *Norovirus* and the family *Caliciviridae* causes acute gastroenteritis in humans^1^. NoV shows strong infectivity leading to large epidemics of acute gastroenteritis in various countries including Japan^2^,^3^,^4^. Accumulating evidence suggests that approximately 50% of patients with acute gastroenteritis in the winter season in Japan may be due to NoV infection^5^,^6^. In addition, large outbreaks of food poisoning due to the virus have been reported^7^,^8^,^9^. Thus, NoV is a major causative agent of acute viral gastroenteritis in industrial countries as well as other major viral agents such as rotaviruses^7^,^8^,^9^.

NoV is classified into 5 genogroups (genogroups I–V)^1^. Among them, genogroups I and II are detected mainly in humans^1^. The NoV genome encodes 3 open reading frames (ORF), and ORF2 encodes the NoV capsid protein^10^. On the basis of detailed genetic analysis, Kroneman *et al.* showed that NoV GI and GII strains can be classified into 9 and 22 genotypes, respectively^11^.

In general, the capsid protein may be an essential determinant of the antigenicity of the non-enveloped virus^12^. For example, it plays pivotal roles in not only viral adsorption/entry but also leads to the generation of neutralising antibodies^13^,^14^,^15^,^16^. Thus, to control NoV infection, it is important to understand their antigenic variation^13^,^14^,^15^,^16^. NoV evolution has been investigated considerably, but most studies have focused on NoV GII^17^,^18^,^19^.

Recent advances of genetic analysis algorithms enable us to obtain the evolutionary information of various viruses. For example, we can assess the evolutionary time scale of viral genes using the Bayesian Markov chain Monte Carlo (MCMC) method^20^. In addition, maximum likelihood approaches may enable us to analyse the determinants of adaptation in viral proteins such as NoV capsid protein^17^,^18^. In the present study, we utilise these methods to analyse comprehensively the molecular evolution of the NoV GI capsid gene.

## Results

### Phylogenetic analysis and evolutionary rates of the NoV capsid gene by the Bayesian MCMC method

**Figure 1:**
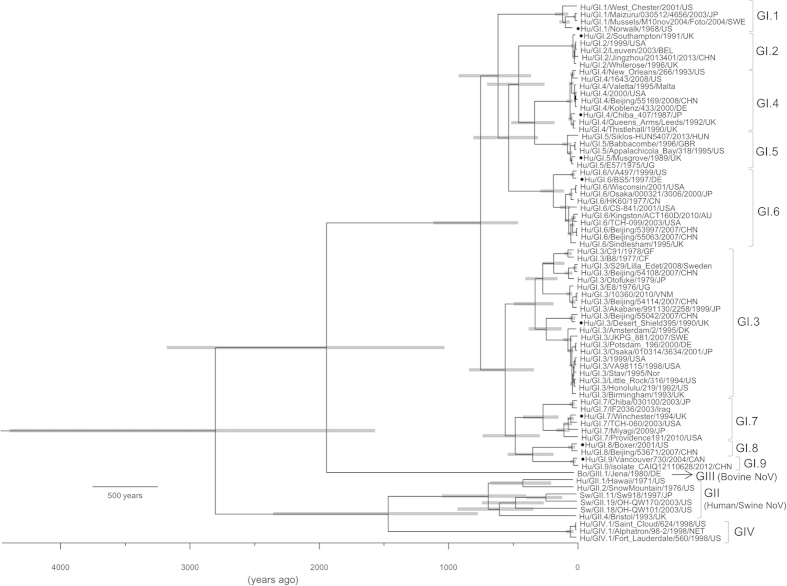
Phylogenetic tree of ORF2 constructed by the Bayesian Markov Chain Monte Carlo method. The phylogenetic tree was based on the whole nucleotide sequence of ORF2 (1593 nt corresponding to GI.1/Norwalk/1968/US). We analysed 65 strains of GI, 6 strains of GII, 1 strain of GIII, and 3 strains of GIV. Each node represents mean root height. The scale bar represents the unit of time (years). The grey bars indicate the 95% HPDs for the estimated year. The reference strains of each genotype are indicated by solid circles.

We constructed a phylogenetic tree with an evolutionary time scale by the Bayesian MCMC method. The 95% highest posterior densities (HPDs) for each node of the phylogenetic tree are indicated by grey bars in Fig. 1. In the present phylogenetic tree, the NoV GI strains divided into 2 lineages about 750 years ago. These lineages are subdivided into 9 genotypes (genotypes 1–9). Lineage 1 contains genotypes 1, 2, 4, 5, and 6, while lineage 2 contains genotypes 3, 7, 8, and 9. Furthermore, genotype 2, 4, 5, 6, and genotypes 7–9 subdivided from the same ancestor virus, while genotype 1/genotype 3 evolved uniquely. The mean evolutionary rate of the present strains was estimated as 1.26 × 10^−3^ substitutions/site/year (95% highest posterior density [HPD] 7.22 × 10^−4^–1.79 × 10^−3^). In addition, we obtained the evolutionary rate of 5 genotypes (GI.2–GI.6), while the rate could not be obtained for the other 4 genotypes due to the small number of strains analysed (Supplemental Table S1). As a result, the evolutionary rate of them was significantly different (*p* < 0.05, Kruskal-Wallis test). These results suggested that an ancestor NoV GI strain diverged from the ancestor of NoV GII, GIII, and GIV strains and it could be dated back to 1570–4390 years ago, corresponding to 95% HPD (mean diverged year, 2803 years ago) (Fig. 1). Furthermore, the present NoV GI strains diverged about 750 years ago and the virus formed 9 genotypes with wide genetic divergence and rapid evolution.

### Selection pressure analysis

To estimate comprehensively the positive selection sites in the capsid protein of NoV, we used 4 methods: conservative single likelihood ancestor counting (SLAC), fixed effects likelihood (FEL), internal fixed effects likelihood (IFEL), and mixed effects model of evolution (MEME) (Table 1). Only 2 positive selection sites were estimated by the FEL and IFEL methods, while 19 sites were estimated using MEME. Notably, the amino acid (aa) substitutions of aa10 consisted of a variety of amino acids. In addition, the substitutions of aa557 were of a single amino acid. However, these substitution sites were not located in the protruding 2 (P2) domain, which is associated with cellular binding site of the capsid protein for NoV infection. Furthermore, over 400 negative selection sites were found in the capsid gene (Table 2). These results suggested that the positive selection sites in the NoV GI capsid protein are located mainly near the N- and C-terminal regions.

### Predicted epitopes in reference strains

Using multiple methods such as LEPS^21^, BCPRED^22^, FBCPRED^22^, BepiPred^23^, Antigenic^24^, and LBtope^25^, we predicted the B-cell linear epitopes in the deduced amino acid sequences of the NoV capsid protein in the reference strains. In the present study, we accepted the epitopes as those identified with 4 or more methods and with >10 consecutive amino acids^26^. The detailed data are shown in Table 3. Many epitopes were estimated in the capsid protein of each NoV GI genotype. Of them, an epitope of GI.1 (aa377–388) may be associated with the histo-blood group antigen (HBGA) binding sites (Ser377, Pro378, and Ser380)^27^ (Table 3). In addition, 1–3 epitopes in each genotype were found in the P2 domain. In the present GI strains, a consensus epitope motif, PAPxGFP, was predicted in the P2 domain in 7 of the 9 genotypes (GI.1, 3–7, and 9). These results suggested that a few viral binding sites of host cells are linked to the epitope sites in the capsid protein of the viruses.

### Phylodynamics of NoV GI strains

**Figure 2:**
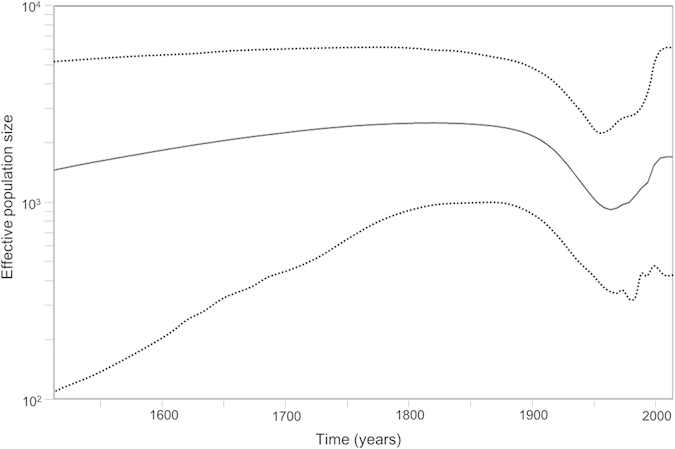
Bayesian skyline plot of ORF2 in NoV GI. The Bayesian skyline plot was estimated under the GTR-Γ_4_ model. The MCMC chains were run for 65,000,000 steps. The Y-axis represents the effective population size and the X-axis represents generation time (year). The solid black line represents the mean value over time. The 95% HPD intervals are shown in dotted lines.

We assessed the phylodynamics of the capsid gene of the NoV GI strains using Bayesian skyline plot (BSP) analysis. As a result, the present strains showed effective population size values over 1000 for a period of 500 years (Fig. 2). In addition, a relatively constant value was seen from 1500–1900 CE, but thereafter the values tended to be low (Fig. 2). These results suggested that NoV GI strains might have adapted to humans over 500 years ago.

### Pairwise distance values of intergenogroup and intergenotypes

**Figure 3:**
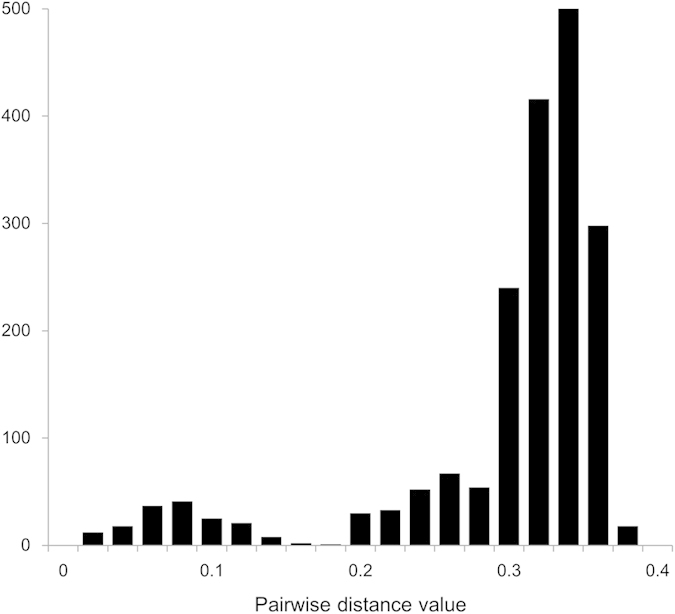
Distributions of the pairwise distance values of ORF2. The distributions of the *p*-distance values based on the nucleotide sequences of NoV GI. A total of 65 strains were analysed.

To assess the genetic distance among the present strains, we calculated their pairwise distance (*p*-distance) (Fig. 3). The *p*-distance value of the intergenogroup was 0.29 ± 0.07 (mean±standard deviation [SD]). The *p*-distance value of the intergenotypes was 0.036 ± 0.010–0.192 ± 0.082 (mean ± SD). These results suggested that the NoV GI capsid gene has undergone considerable genetic divergence (intergenogroup *p*-distance > 0.25).

## Discussion

We studied the molecular evolution of the capsid gene in NoV genogroup I. First, we found that the human NoV GI strains diverged approximately 2,800 years ago from the ancestor of the GII, GIII, and GIV strains, although the mean estimated time of divergence had a large variation (95% HPD, 1570–4390 years ago). NoV GI evolved rapidly (approximately 10^−3^ substitutions/site/year). They also had wide genetic divergence (*p*-distance > 0.25). In addition, the NoV GI strains diverged and formed 9 genotypes over a period of about 750 years. Some genotypes (genotypes 2, 4, 5, and 6) evolved from the same ancestor. Second, 2–19 positive selection sites and over 400 negative selection sites were estimated in the deduced capsid protein of NoV GI. Third, many epitopes were estimated in the deduced capsid proteins. However, there were few epitopes at the cellular binding site of the capsid protein. Furthermore, BSP analysis suggested that NoV GI strains adapted to humans a long time ago.

With regard to norovirus, many evolutionary and/or molecular epidemiological studies have been reported^17^,^18^,^19^,^28^. For example, Rackoff *et al.*, estimated the molecular evolution rate of NoV GI.1 and GI.3 as 1.37 × 10^−3^ and 1.25 × 10^−3^ substitutions/site/year, respectively^28^. In the present study, we obtained a mean evolutionary rate of 1.26 × 10^−3^ substitutions/site/year for all NoV GI genotypes. The evolutionary rate among the genotypes GI.2–GI.6 were significantly different (Supplemental Table S1). These results suggested that the evolutionary rate of the NoV GI capsid gene was variable among NoV GI genotypes, although these data, including our own, are limited, because they were estimated using a small number of strains. However, the present study may be the first report to estimate the evolutionary rate for all genotypes of the NoV GI capsid gene.

The capsid protein of a non-enveloped virus plays pivotal functions such as adsorption and entry of the target cells^12^. Divergence of the capsid protein may be linked to the antigenicity of various viruses^12^. Thus, divergence of the capsid protein may reflect differences in the antigenicity of NoV. Furthermore, host defence mechanisms, including the immune system, may act as a selective pressure to NoV. In general, a viral protein with strong antigenicity may undergo strong selection pressure, resulting in the presence of many positive selection sites in the antigenic protein^29^. Indeed, many positive selection sites were found in the capsid proteins of an enterovirus showing strong antigenicity^30^. To date, some representative studies regarding the relationship between positive selection and antigenicity in NoV have been reported^17^,^28^. For example, Cotton *et al.* showed some positive selection sites in NoV GII strains, at Glu106Arg and Asn298Asp^31^. Moreover, Siebenga *et al.* confirmed some sites in NoV GII/4: Asn6Ser, Asn9Ser/Thr, Ala15Thr, Ile47Val, and Ala534Thr/Val^17^. The capsid proteins of NoV GI and GII may have undergone selective pressure mainly near the N- and C-terminal regions in the host. In the present study, variations (at 2–19 sites) among the 4 models—SLAC, FEL, IFEL, and MEME—was found. In each method, differences in the number of positive selection sites have been found in other virus genomes^32^,^33^. This may be due to differences in the principles used in each method to estimate the sites^34^,^35^.

Two distinct types of epitope, T-cell-recognised and B-cell-recognised epitopes, have been confirmed^36^. B-cell-recognised epitopes may be an important index for the prediction of antibody binding sites against NoV GI. Next, previous reports suggested that the HBGA binding sites of the viral P2 domains are associated with infection of host cells^27^. In the present study, although we only estimated B-cell linear epitopes, we found the following: 1) many predicted epitopes were found in the capsid protein of NoV GI; and 2) a consensus epitope motif (PAPxGFP) was estimated in 7 of the 9 GI genotypes (Table 3). Regarding the HBGA binding sites, many predicted epitopes were found in the capsid protein of NoV GI. Among them, an epitope of GI.1 (aa377–388) was estimated at an HBGA binding site of the P2 domain (Table 3)^27^. Previous reports showed that the host cellular binding sites of NoV may be located in the P2 domain of the capsid protein (corresponding to aa279–405 in ORF2 of GI.1/Norwalk/1968/US)^37^,^38^,^39^. If epitopes are located in the P2 domain, the immune system may react with them, leading to the generation of a neutralising antibody. Furthermore, previous reports estimated some epitopes in NoV GII strains^40^. These epitopes are located in the P2 domain on the surface of the capsid protein of GII.4 strains^40^. Furthermore, some positive selection sites were identified in an area of the P2 domain associated with blockade epitope A by using a monoclonal antibody^31^. An effective neutralising antibody may inhibit NoV infection of the host; however, the majority of epitopes in the other NoV GI genotypes were not detected at the HBGA binding sites of the P2 domain. However, we did not confirm the conformational epitopes in the present NoV GI strains. In various RNA viruses, such as dengue viruses, the conformational epitopes may be associated with the production of neutralising antibodies^41^,^42^,^43^. Together, further studies regarding the relationships among B-cell epitopes, including the conformational epitopes, HBGA binding sites, and the consensus epitope motif (PAPxGFP), are needed to assess whether the human immune system can produce effective neutralising antibodies against most types of NoV GI.

Next, to evaluate the effective population size of NoV GI, we performed BSP analysis. This method enables us to estimate the effective population size over a period of several hundred years, even in there are no sequences from strains aged more than 50 years^20^. The effective population size showed a constant value from 1500 to 1900 CE. The values decreased from 1900 to 1950 CE; however, after that, the values were restored. With regard to NoV GII.4, no relationship was found between epidemics of the virus and its effective population size based on calculations using the capsid gene^17^. Conversely, a relationship was found between epidemics of the virus and its effective population size based on partial sequencing of the polymerase gene^17^. Both previous and present results suggest that NoV GI strains evolved and maintained constant genetic divergence. The virus may be subject to selection pressure in the host, resulting in the lack of a significant change in its effective population size.

In conclusion, NoV GI strains evolved rapidly and their common ancestor dated back to approximately 750 years ago. The virus may be under local positive selection to escape the immune system of the host, resulting in its adaptation to humans. In addition, to understand better the molecular evolution of NoV GI, further studies regarding the evolution of other genes including the RNA dependent RNA polymerase (RdRp) gene may be needed.

## Materials and Methods

### Strains and alignments

We collected complete capsid gene (ORF2) sequences of all NoV GI strains, excluding ORF1/2 recombinant strains, from GenBank. We confirmed the genotype of each strain using the norovirus typing tool NoroNet^44^. All sequences were aligned using Clustal W^45^. More than 99% of identical sequences were removed from the dataset. A total of 65 strains were collected. The nucleotide sequences correspond to positions 1–1593 in ORF2 of GI.1/Norwalk/1968/US (GenBank accession No. M87661). The detailed data are shown in Supplemental Table S2.

### Phylogenetic analysis by the Bayesian Markov Chain Monte Carlo method

We estimated time-scaled phylogeny and evolutionary rate of ORF2 using the Bayesian MCMC method in the BEAST program v1.7.5^20^. To estimate the time of divergence from the other genogroups, some sequences of GII, GIII, and GIV were added to the sequences from 65 GI strains (Supplemental Table S2). The KAKUSAN4 program was used to select the best nucleotide substitution model^46^. Four clock models (strict, lognormal, exponential, and random) and 4 demographic models (constant size, exponential growth, expansion growth and lognormal growth) were compared using Akaike’s information criterion through MCMC (AICM)^47^,^48^. The datasets were analysed using the GTR-Γ_4_ model of substitution under a lognormal relaxed clock model with an exponential growth model. The MCMC chains were run for 50,000,000 steps to achieve convergence with sampling every 1000 steps. Convergence was assessed from the effective sample size (ESS) after a 10% burn-in using Tracer v1.6^49^. Only parameters with an ESS above 200 were accepted. Uncertainty in the estimates was indicated by the 95% HPD intervals. The maximum clade credibility tree was generated by Tree Annotator v 1.7.5 after a 10% burn-in. The phylogenetic tree was viewed in FigTree v1.3.1. The evolutionary rates of each genotype were also calculated. In addition, to estimate changes in the effective population size through time of NoV GI, a BSP was constructed using the BEAST program as described above.

### Statistical analyses

Statistical analyses were performed using the Kruskal-Wallis test or Mann-Whitney U test with Bonferroni correction using EZR^50^. Values of *p* < 0.05 were considered to be significant.

### Estimation of positive and negative selection sites

To evaluate the selection pressure on the ORF2 region, synonymous (*d*S) and nonsynonymous (*d*N) substitution rates at every codon were calculated by Datamonkey using the following methods: SLAC, FEL, IFEL, and MEME^51^. SLAC is intensive for large alignments compared to the other methods^35^; however, this method pretends to underrate the substitution rate^35^. In contrast, the FEL and IFEL methods consider both synonymous and nonsynonymous rate variations and may be efficiently parallelised^35^. MEME can consider episodic selective pressure^34^. We employed 4 different methods for accurate calculations. The cut-off *p*-value was set at 0.05.

### Epitope prediction

The B-cell linear epitopes of the standard reference strains were predicted as described previously^26^. We used the following six tools: LEPS^21^, BCPRED^22^, FBCPRED^22^, BepiPred^23^, Antigenic^24^, and LBtope^25^. All tools were used in the default condition. We accepted the common epitopes estimated by 4 or more tools and with >10 consecutive amino acids^26^.

### Calculation of *p*-distance values

To assess the frequency distribution of NoV GI, the *p*-distance values of intergenogroup and intergenotypes were calculated. We analysed the present strains using MEGA 6.0^52^.

## Additional Information

**How to cite this article**: Kobayashi, M. *et al.* Molecular Evolution of the Capsid Gene in Norovirus Genogroup I. *Sci. Rep.* **5**, 13806; doi: 10.1038/srep13806 (2015).

## Supplementary Material

Supplementary Information

## Figures and Tables

**Table 1:** Positive selection sites in ORF2 of NoV GI.

aa change	SLAC	FEL	IFEL	MEME
Met2Lys				○
Asp7Gly				○
Pro10Thr, Gln, Thr, SerThr10SerSer10Pro, Thr		○	○	○
Ser16Thr, Asn				○
Ala18Arg				○
Gly19Arg				○
Ile354Val				○
Asn358Asp, LeuAsp358Ser, AsnSer358Asp				○
Val359Ile, MetIle359Val				○
Ala367Val				○
Ile390Thr, Leu, Val, Met				○
Lei393Ile				○
Ser397Cys, Leu				○
Ser400Thr, ArgThr400Ser, Ala, Glu				○
Asp401Asn, Ser, Gly, IleAsn401Ser				○
Ala402Pro, Ser, Val,Gly, AspSer402Asn				○
Val409Asp, Ile, AlaAsp409Asn				○
Ala545Thr, Arg				○
Ser557LeuLeu557IleVal557Ile		○	○	○

Cut-off value, *p* < 0.05.

**Table 2:** Negative selection sites in ORF2 of NoV GI.

	SLAC	FEL	IFEL
No. of negative selection sites	437	469	428

Cut-off value, *p* < 0.05.

**Table 3:** Predicted epitopes of the reference strains for each genotype.

Genotype	Strain (GenBank accession No.)	Position	Predicted epitopes
GI.1	Norwalk/1968/US (M87661)	32–43	AMDPVAGSSTAV
		316–325	APIGFPDLGG
		377–388	**SPPSHPSGSQVD**
		480–496	FLTCVPNGASSGPQQLP
GI.2	Southampton/1991/UK (L07418)	33–45	MEPVAGPTTAVAT
		413–423	**AANL**APPVFPP
		437–448	PGPNNRSAPNDV
		501–511	NGVGAGPQQLP
GI.3	Desert Shield395/1990/UK (U04469)	314–326	**YHAFESPAPIGFP**
GI.4	Chiba 407/1987/JP (AB022679)	29–43	DPIPIDPVAGSSTAL
		157–167	PVEVPLEDVRN
		317–327	**APAPAGFPDLG**
		386–397	**TSPPSDSGGANT**
		435–445	IPGPNQSGSPN
		501–510	SSSTGPQQLP
		529–539	PVGTAGPARGR
GI.5	Musgrove/1989/UK (AJ277614)	9–19	TPSADGANGAG
		29–41	EPLPLDPVAGAST
		319–329	**APTGFPDLGTS**
		436–447	IPGPNTAHKPNL
GI.6	BS5/1997/DE (AF093797)	312–325	**PFVPLESPAPVGFP**
GI.7	Winchester/1994/UK (AJ277609)	28–41	AEPLPLEPVVGAAT
		189–200	LRAGGASSGTDP
		314–326	**YHAFESPAPLGFP**
		392–402	**GARVDPWKIPS**
		495–506	PNTGGGPQNLPT
GI.8	Boxer/2001/US (AF538679)	189–200	LRSGGASSGTDP
		342–353	**PTELSTGDPSGK**
		441–453	TVSNPKVPCTLPQ
		499–509	PNAGGGPQTLP
GI.9	Vancouver730/2004/CAN (HQ637267)	20–33	QLVPENNNTSEPIN
		318–334	**HAFESPAPLGFPDFGDG**
		351–366	**NDPVVVGNVQPYNPQF**
		374–385	**VVENPTPDQVAT**

The predicted epitopes in the P2 domain are indicated by bold type and underlined.

Common epitopes are indicated by grey shading.
