# Supplementary material for: Molecular Evolution of the Capsid Gene in Norovirus Genogroup I
Source: Sci Rep. 2015 Sep 4;5:13806. doi: 10.1038/srep13806 (PMC4559769; doi:10.1038/srep13806)
Supplement: Supplementary Information [file srep13806-s1.pdf]

Molecular Evolution of the Capsid Gene in Norovirus Genogroup I

Miho Kobayashi, Shima Yoshizumi, Sayaka Kogawa, Tomoko Takahashi, Yo Ueki, Michiyo Shinohara, Fuminori Mizukoshi, Hiroyuki Tsukagoshi, Yoshiko Sasaki, Rieko Suzuki, Hideaki Shimizu, Akira Iwakiri, Nobuhiko Okabe, Komei Shirabe, Hiroto Shinomiya, Kunihisa Kozawa, Hideki Kusunoki, Akihide Ryo, Makoto Kuroda, Kazuhiko Katayama, and Hirokazu Kimura*

*E-mail: kimhiro@nih.go.jp

Supplementary Information:
Mean evolutionary rates are shown in Table S1
We added detailed data for all NoV strains used in the present study as Table S2.

Table S1. Mean evolutionary rates of each genotype.

Genotype	Mean evolutionary rate (substitutions/site/year)	Substitution model	Clock model	Demographic model	MCMC chain length*
GI.1 (4 strains)	not conpatible				
GI.2 (5 strains)	1.88×10^{-3}	HKY85-Γ	Random clock	Logistic growth	15,000,000
GI.3 (21 strains)	3.28×10^{-3}	GTR-Γ	Exponential clock	Constant size	50,000,000
GI.4 (9 strains)	2.68×10^{-3}	HKY85-Γ	Exponential clock	Expansion growth	12,000,000
GI.5 (5 strains)	3.17×10^{-3}	GTR-Γ	Exponential clock	Exponential growth	700,000,000
GI.6 (11 strains)	1.66×10^{-3}	GTR-Γ	Random clock	Expansion growth	10,000,000
GI.7 (6 strains)	not conpatible				
GI.8 (2 strains)	not tested				
GI.9 (2 strains)	not tested				

*Sampling every 1000 steps.

Table S2. Strains used in this study.

GenBank accession no.	Genotype	Collection year	Strain
M87661	GI.1	1968	Hu/GI.1/Norwalk/1968/US
AY502016	GI.1	2001	Hu/GI.1/West Chester/2001/USA
EF547392	GI.1	2003	Hu/GI.1/Maizuru/030512/4656/2003/JP
EU085522	GI.1	2004	Hu/GI.1/Mussels/M10nov2004/Foto/2004/SWE
L07418	GI.2	1991	Hu/GI.2/Southampton/1991/UK
JQ743332	GI.2	1999	Hu/GI.2/1999/USA
J277610	GI.2	1996	Hu/GI.2/Whiterose/1996/UK
FJ515294	GI.2	2003	Hu/GI.2/Leuven/2003/BEL
KF306212	GI.2	2013	Hu/GI.2/Jingzhou/2013401/2013/CHN
U04469	GI.3	1990	Hu/GI.3/Desert Shield395/1990/UK
FJ711163	GI.3	2007	Hu/GI.3/JKPG 881/2007/SWE
AF414403	GI.3	1992	Hu/GI.3/Honolulu/219/1992/US
AF414405	GI.3	1994	Hu/GI.3/Little Rock/316/1994/US
KJ194510	GI.3	1995	Hu/GI.3/Amsterdam/2/1995/DK
AB187514	GI.3	1979	Hu/GI.3/Otofuke/1979/JP
AF145709	GI.3	1995	Hu/GI.3/Stav/1995/Nor
AJ277612	GI.3	1993	Hu/GI.3/Birmingham/1993/UK
AF439267	GI.3	2000	Hu/GI.3/Potsdam 196/2000/DE
GQ856470	GI.3	2007	Hu/GI.3/Beijing/54108/2007/CHN
GQ856473	GI.3	2007	Hu/GI.3/Beijing/55042/2007/CHN
JN603244	GI.3	2008	Hu/GI.3/S29/Lilla Edet/2008/Sweden
JN699048	GI.3	1978	Hu/GI.3/C91/1978/GF
JN699050	GI.3	1977	Hu/GI.3/B8/1977/CF
EF547396	GI.3	1999	Hu/GI.3/Akabane/991130/2258/1999/JP
GQ856471	GI.3	2007	Hu/GI.3/Beijing/54114/2007/CHN
JN699047	GI.3	1976	Hu/GI.3/E8/1976/UG
JQ911594	GI.3	2010	Hu/GI.3/10360/2010/VNM
EF547393	GI.3	2001	Hu/GI.3/Osaka/010314/3634/2001/JP
JQ743330	GI.3	1999	Hu/GI.3/1999/USA
AY038598	GI.3	1998	Hu/GI.3/VA98115/1998/USA
AB022679	GI.4	1987	Hu/GI.4/Chiba 407/1987/JP
AF394960	GI.4	2000	Hu/GI.4/Koblenz/433/2000/DE
AF414402	GI.4	1993	Hu/GI.4/New Orleans/266/1993/US
GQ413970	GI.4	2008	Hu/GI.4/1643/2008/US
GQ856475	GI.4	2008	Hu/GI.4/Beijing/55169/2008/CHN
AJ277616	GI.4	1995	Hu/GI.4/Valetta/1995/Malta
JQ743331	GI.4	2000	Hu/GI.4/2000/USA
AJ277621	GI.4	1990	Hu/GI.4/Thistlehall/1990/UK
AJ313030	GI.4	1992	Hu/GI.4/Queens Arms/Leeds/1992/UK
AJ277614	GI.5	1989	Hu/GI.5/Musgrove/1989/UK
KJ402295	GI.5	2013	Hu/GI.5/Siklos-HUN5407/2013/HUN
AF414406	GI.5	1995	Hu/GI.5/Appalachicola Bay/318/1995/US
AM263418	GI.5	1996	Hu/GI.5/Babbacombe/1996/GBR
JN699046	GI.5	1975	Hu/GI.5/E57/1975/UG
AF093797	GI.6	1997	Hu/GI.6/BS5/1997/DE
AY502007	GI.6	2001	Hu/GI.6/CS-841/2001/USA
GQ856463	GI.6	2007	Hu/GI.6/Beijing/53997/2007/CHN
GQ856464	GI.6	2007	Hu/GI.6/Beijing/55063/2007/CHN
JN699045	GI.6	1977	Hu/GI.6/HK60/1977/CN
JQ388274	GI.6	2010	Hu/GI.6/Kingston/ACT160D/2010/AU
EF547395	GI.6	2000	Hu/GI.6/Osaka/000321/3006/2000/JP
AF538678	GI.6	1999	Hu/GI.6/VA497/1999/US
KC998959	GI.6	2003	Hu/GI.6/TCH-099/2003/USA
AJ277615	GI.6	1995	Hu/GI.6/Sindlesham/1995/UK
AY502008	GI.6	2001	Hu/GI.6/Wisconsin/2001/USA
AJ277609	GI.7	1994	Hu/GI.7/Winchester/1994/UK
JN899243	GI.7	2010	Hu/GI.7/Providence191/2010/USA
JN005886	GI.7	2003	Hu/GI.7/TCH-060/2003/USA
AY675555	GI.7	2003	Hu/GI.7/IF2036/2003/Iraq
AJ844469	GI.7	2003	Hu/GI.7/Chiba/030100/2003/JP
AB758449	GI.7	2009	Hu/GI.7/Miyagi/2009/JP
AF538679	GI.8	2001	Hu/GI.8/Boxer/2001/US
GQ856462	GI.8	2007	Hu/GI.8/Beijing/53671/2007/CHN
HQ637267	GI.9	2004	Hu/GI.9/Vancouver730/2004/CAN
KF586507	GI.9	2012	Sw/GI.9/isolate CAIQ12110628/2012/CHN
U07611	GII.1	1971	Hu/GII.1/Hawaii/1971/US
U70059	GII.2	1976	Hu/GII.2/SnowMountain/1976/US
X76716	GII.4	1993	Hu/GII.4/Bristol/1993/UK
AB074893	GII.11	1997	Sw/GII.11/Sw918/1997/JP
AY823304	GII.18	2003	Sw/GII.18/OH-QW101/2003/US
AY823306	GII.19	2003	Sw/GII.19/OH-QW170/2003/US
AJ011099	GIII.1	1980	Bo/GIII.1/Jena/1980/DE
AF195847	GIV.1	1998	Hu/GIV.1/Alphatron/98-2/1998/NET
AF414427	GIV.1	1998	Hu/GIV.1/Saint Cloud/624/1998/US
AF414426	GIV.1	1998	Hu/GIV.1/Fort Lauderdale/560/1998/US
